# High workload and job stress are associated with lower practice performance in general practice: an observational study in 239 general practices in the Netherlands

**DOI:** 10.1186/1472-6963-9-118

**Published:** 2009-07-15

**Authors:** Pieter van den Hombergh, Beat Künzi, Glyn Elwyn, Jan van Doremalen, Reinier Akkermans, Richard Grol, Michel Wensing

**Affiliations:** 1 IQ Healthcare, Center for Quality of Care Research, University of Nijmegen, PO Box 9101, 6500 HB Nijmegen, the Netherlands; 2 Swisspep Institute for Quality and Research in Healthcare, PO Box, CH-3073 Guemligen, Switzerland; 3 Department of Primary Care and Public Health, School of Medicine, Cardiff University, Cardiff, CF14 4YS, Wales, UK; 4 Department of General Practice and Health Services Research, Heidelberg University Hospital, Germany

## Abstract

**Background:**

The impact of high physician workload and job stress on quality and outcomes of healthcare delivery is not clear. Our study explored whether high workload and job stress were associated with lower performance in general practices in the Netherlands.

**Methods:**

Secondary analysis of data from 239 general practices, collected in practice visits between 2003 to 2006 in the Netherlands using a comprehensive set of measures of practice management. Data were collected by a practice visitor, a trained non-physician observer using patients questionnaires, doctors and staff. For this study we selected five measures of practice performance as outcomes and six measures of GP workload and job stress as predictors. A total of 79 indicators were used out of the 303 available indicators. Random coefficient regression models were applied to examine associations.

**Results and discussion:**

Workload and job stress are associated with practice performance.

*Workload*: Working more hours as a GP was associated with more positive patient experiences of accessibility and availability (b = 0.16). After list size adjustment, practices with more GP-time per patient scored higher on GP care (b = 0.45). When GPs provided more than 20 hours per week per 1000 patients, patients scored over 80% on the Europep questionnaire for quality of GP care.

*Job stress*: High GP job stress was associated with lower accessibility and availability (b = 0.21) and insufficient practice management (b = 0.25). Higher GP commitment and more satisfaction with the job was associated with more prevention and disease management (b = 0.35).

**Conclusion:**

Providing more time in the practice, and more time per patient and experiencing less job stress are all associated with perceptions by patients of better care and better practice performance. Workload and job stress should be assessed by using list size adjusted data in order to realise better quality of care. Organisational development using this kind of data feedback could benefit both patients and GP.

## Background

High workload and work-related stress are known to increase the risk of alcohol and drug abuse, problems in social relationships, depression and anxiety, and suicide in doctors.[1] However, the impact of high physician workload and job stress on quality and outcomes of healthcare delivery is less clear. High workload and job stress may have negative impact on practice performance and increase the risk for occupational health hazards.[2] Insights into the underlying mechanisms and the size of such effects are limited. Most research has been on consultation length and the quality of the consultation. Wilson et al. (2002) make the point that it cannot be shown whether consultation length itself is the important variable, or whether it is simply a marker for other attributes of the doctor, e.g. female GPs have longer consultations, give more lifestyle advice, prescribe less, recognized more psychosocial problems and did more examinations. Wilson and Child's (2002) are clear about previous studies: "Only four studies (out of a selection of 10 studies out of 42 papers) examined outcome measures. In two studies, there were differences in enablement and in satisfaction with consultation duration, but not overall satisfaction, suggesting that average consultation length may be associated with some better short-term outcomes. This review illustrates the need to explore relationships between average consultation length and clinical outcomes, such as control of chronic disease".[3]

Such knowledge is needed to determine the need for interventions in a practice targeted at physician workload and job stress, and to tailor such interventions to the most relevant factors.

The aim of this study was to identify and quantify associations between practice performance and measures of workload and job stress in general practice.

## Methods

### Design and Study population

We performed an exploratory secondary analysis of data collected from 239 general practices in The Netherlands. These practices were included between 2003 and 2006 as part of a voluntary, ongoing service for practice visits provided by the Dutch College of General Practitioners (NHG). In 2003 the Europep questionnaire was added to the VIP (Visit Instrument Practice Management). In table 1 we compare the characteristics of the visited practices with national data.[4] In this cross sectional correlational analysis, measures of GP workload and job stress were analysed for their relation with measures of quality of care. The study was done before The Netherlands changed from a capitation payment to a system also based on fee for services and targets.

The ethical committee Arnhem-Nijmegen stated that ethical approval was not required for this project.

**Table 1:** Practices in our study (n = 239) compared to the Dutch national study (n = 4533)

**Characteristics of the practice**	**Practices in our study (n = 239)**	**Dutch Practices (n = 4533)#**
Practice setting		
*Single-handed*	110 (48.5%)	2248 (49.6%)
*Two partners*	59 (26.0%)	1369 (30.2%)
*Group practice*	58 (25.5%)	916 (20.2%)

Rural practices of total	31 (13.3%)	444 (9.8%)
Fte GP/1000 patients	0.41 fte	0.42 fte

Characteristics of GPs	N = 546	**N = 8408**

Percentage < 45 years	231 (42.3%)	3018 (36%)
Sex (female)	215 (39.5%)	2773 (33%)
Full-time	179 (32.8%)	4481 (53)%

# Figures on 4533 practices and 8408 GP in the Netherlands (NIVEL, 2005; )

### Measures

In all practices a comprehensive measurement of practice management was performed, using the previously validated instruments VIP and Europep.[5] The measures are based on questionnaires for GPs, practice nurses, on structured observation by practice visitors and, on results of patient questionnaires (30 patients per GP and 30 patients per practice).[6] The VIP is an assessment method developed, tested, validated and continuously revised since 1995 and used by over 2500 GPs in 1500 practices to audit their practice management.[7,8] For a completed visit all questionnaires had to be completed. The VIP has 303 indicators, which can be clustered into 59 dimensions or four large areas of practice management i.e.: Infrastructure (premises, equipment, service and organisation); Team (task division, workload and job stress of the GPs); Communication (with colleagues/care providers, meeting time, patient information, computerised patient records, IT) and Quality Improvement (CME, audit, QA-activities). The Europep is an internationally validated and widely used questionnaire for patient experience of general practice.[9] For this study we selected five out of the 59 dimensions on practice performance, three of GP workload and three of job stress.

Practice performance was operationalised in five specific composite measures, which reflected both patient experiences and the presence of specific items of care provision. Each of the measures was based on a number of indicators. Principal component and internal consistency analyses were used to reconfirm the previously established internal consistency of the measures.

The following five composite measures were used for practice performance (see table 2):

**Table 2:** Descriptive data on measures of practice performance, workload and job stress (N = 239)

**Measures of practice performance + N of questions**	**Mean + SD**	**Range**	**Cronbach's α**
Patient experience with accessibility & availability (5)	78.6% ± 10.4	25.6 – 95.6	0.72

Patient experience with organisation of surgery (6)	86.0% ± 6.9	66.8 – 98.2	0.72

Patient opinion on practice management (6)	67.8% ± 11.1	38.8 – 98.2	0.96

Patient opinion on GP care (17)	80.4% ± 7.6	55.2 – 97.1	0.88

Prevention and disease management (11 indicators)	7.0 ± 3.0	0 – 11	0.84

**Workload (hours)**	**Mean + SD**		

Total workload as a GP = *Total number of hours/week working professionally*	46.9 hours ± 9.4	27.5 – 77.5	

Actual GP-time per patient = *Hrs/wk on the job/1000 patients (including administration & organisation)*	21.7 hours ± 4.8	11.6 – 41.3	

Proportion of time spent on patient care = *Hrs/wk of direct patient care/Total of hrs/wk working*	0.57 ± 0.09	0.29 – 0.83	

**Job stress + N of questions**	**Mean + SD**		**Cronbach's α**

Experience of inappropriate patient demands (4)	13.2 ± 2.1	5 – 19	0.67

Higher commitment and satisfaction with the job (4)	6.8 ± 1.4	3 – 13	0.72

Lower experienced workload (16)	69.0 ± 7.1	34 – 80	0.93

1. Patient experience with accessibility and availability (patient approves – of emergency service during office hours, – of information on practice regulations, – of on call arrangements, – of accessibility by telephone in emergencies, – patient prefers practice over emergency service of hospital; 5 indicators).

2. Patient experience with the organisation of surgery (patient finds the surgery hours appropriate, can get a consultation at a convenient time, can get extra consultation time, can easily consult his own GP by telephone, does not experience the assistant as a hurdle to contact the GP, does not regularly get a different GP; 6 indicators).

3. Patient evaluation of practice management (Europep; the patient opinion on the practice organisation (helpfulness staff, suitable appointment, waiting time, quick service, telephone access to GP/practice); 6 indicators).

4. Patient evaluation of GP performance. (Europep; The patient opinion on the quality of interpersonal care; 17 indicators).

5. Prevention and Disease Management (DM, CVD, Asthma/COPD, PAP-smear, life style advice; 11 indicators).

GP workload was operationalised in three composite measures.

1. Total GP workload (not corrected for list size): Total number of hours per week working as a GP 'including all other professional activities (e.g. teaching, research).

2. Proportion of time spent on patient care: Number of hours the GP spends on direct patient care (consultations, home visits, telephone calls)/Total GP workload (as in 1).

3. 'Time per patient'. Number of hours of GP-care per week per 1000 patients (including administration, organisation, worked hours being on call and CME-hours)

GP job stress was operationalised in three composite measures [10]:

1. GP's experience of inappropriate patient demands (4 indicators) Questions are "I get the feeling that some aspects of my job do not really make sense"; "My work consists of many unnecessary activities; "The media stimulate inappropriate patient demand"; "I spend too much time on illnesses that don't require medical attention".

2. Commitment with the job (4 indicators); the GP is committed and interested in his work and likes the job.

3. Experienced workload (16 indicators); Physical symptoms of job stress; being tired after work, concentration, etc.

### Data-analysis

**Figure 1:**
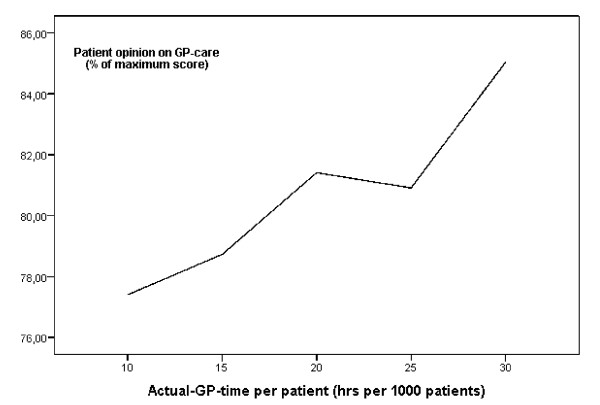
**Actual GP time per 1000 patients and patient evaluations of GP performance**. 10 hrs 77.4 (N = 11), > 15 hrs 78.7 (N = 82), > 20 hrs 81.4 (N = 92), >25 80.9 (N = 43), > 30 hrs 85.3 (N = 11).

The data of all 239 practice visits were aggregated and analysed at the practice level. A GLM-procedure and stepwise linear regression analysis determined how much variation in the six measures of practice management was associated with the measures of physician workload and job stress. Only measures with significant effects (p < 0.05) entered the final multivariate model. For the analysis we used SAS 9.1 and for the graphical presentation in figure 1 we used SPSS 12 for Windows.

For the power calculation we used a predictive regression model with 6 predictors (alpha = 0.05, power = 80 and a medium effect size (*f*^2 ^= 0.15). This requires a sample size of 97 cases to detect a significant model.[11] Cohen has rated effect sizes of 0.02, 0.13, 0.36 as small medium and large respectively corresponding with *R*^2 ^= 0.02, 0.13, 0.36.[12]

## Results

### Description of practice sample

The sample of 239 practices was representative of general practice in the Netherlands. Rural practices may be slightly overrepresented. The GPs were slightly younger, more often female and working part time than average. (table 1)

### GP Workload

A higher workload as a GP (= working more hours per week professionally or on the job in the practice) resulted in more positive patient experiences of accessibility and availability when uncorrected for list size. (table 3)

**Table 3:** Multivariate analysis of GP workload and job stress as predictors of practice performance; N = 239 practices. Pr > F en CI. Non significant results have been omitted

	**Patient opinion on accessibility/availability**	**Organisation of surgery**	**Europep Patient opinion on practice management**	**Europep Patient opinion on GP care**	**Prevention & Disease Management**
**Workload**	***Estimate, CI***	***Estimate, CI***	***Estimate, CI***	***Estimate, CI***	***Estimate, CI***

Total GP workload = *Hours/week working as a GP*	.16 ± .07* (.020, .295)				
Proportion of time spent on patient care = *Hrs of patient care/Total of hrs/wk working*					
Actual GP-time per patient = *Hrs/wk on the job/1000 patients (incl. organisation)*				.45 ± .12**** (.227, .691)	
**Job stress**					
Experiencing inappropriate demands of patients					
Higher commitment and satisfaction with the job					.35 ± .14 * (-.616, -.076)
Lower experienced workload	.21 ± .09 * (.032, .400)		.25 ± .10 * (.056, .450)		

* p < 0.05 ** p < 0.01 *** p < 0.001 **** p < 0.0001

The time the GP spent on direct patient care (face to face) as a proportion of his/her total workload (working part time in the practice) was not associated with any measure of practice performance. Practices which provided more GP-time per patient had higher scores on the 'patient opinion on GP care'(p < 0.0001). The approximately linear relation is presented in figure 1. Spending roughly more than 20 hours per 1000 patients resulted in a more than 80% score on the patient's evaluation of GP performance.

### GP job stress

Experiencing inappropriate patient demands by the GP was not associated with practice performance. Practices with committed GPs had better organised structured care for chronic patients (p < 0.05).

In practices where GPs experience less job stress, the patient's experience with accessibility and availability (p < 0.05) as well as the organisation of the practice scored higher (p < 0.01).

## Discussion

### Principal Findings

The results of this study indicate that practices where GPs worked for more hours per week, had more time per patient, were more committed or experienced less job stress, all had higher scores on various measures of practice performance. Especially strong was the relation between the 'time per patient' and the patient's opinion on the quality of care. GPs who spent more than average time on patient care scored consistently higher on patient evaluations of GP performance. This measure includes face to face contact as well as the time spent on patients besides consultations.

For a practice of 2000 patients an average GP would have to work on average more than 40 hours to score over 80%. This is the first study to show at the practice level the relationship between the time spent by GPs on patient care and the patient perceptions of practice performance. Previous research focused more on consultation time instead of workload.[3] Studies on workload and job stress focused on the consequences for the physician rather than for the patient or the practice organisation.

### Strengths and limitations of the study

Our study was observational and exploratory. However, it used data from a large sample of general practices and used well-developed validated instruments.[13,14] The sample had slightly more young, female, part-time GPs than the average GP-population in the Netherlands. The study was cross-sectional and therefore the direction of impact cannot be assumed. Our database of practice visits did not yet include information on clinical output (chronic diseases like CVD, DM or asthma/COPD). From 2002 onwards the practice nurse was rapidly introduced in general practice in The Netherlands, increasing performance in disease management. Studies into the effect of the practice nurse showed improved outcome, but no reduction in workload for the GP. Yet, future studies on the practice level should include the workloads of practice nurses. Generalisability of the results to other countries is limited due the size of general practices in the Netherlands. Most practices in France and Belgium are single-handed whereas most practices in the UK, Scandinavia and Australia have larger and multidisciplinary teams.

### Findings in context of other research

The results add weight to previous work.[15] Grol demonstrated poor clinical performance in GPs with negative feelings, lack of time and frustration as evidenced by a high prescription rate and giving little explanation to patients.[16] Stress of GPs is a concern in the UK because of difficulties with retaining the workforce needed to meet the targets of a primary care led NHS.[17,18]. Howie et al. showed that 'fast GPs' with short consultations (< 6 min.) discussed fewer psychosocial problems and prescribed more antibiotics than 'slow GPs' (> 9 min consultations).[19] Faster doctors were less likely than slower doctors to recognize and deal with long-term problems and psychosocial problems.[9] Faster doctors did less preventive activities and also gave less life style advice.[9]

These aspects are part of the Europep questionnaire in our study, thus supporting these results.

Hayter found that low job satisfaction was directly related to lower patient satisfaction and compliance with treatment.[20]. Other factors influencing quality of care are motivation and sleep deprivation. Consultation sessions surrounding nights on call and characterised by anticipatory or hangover stress lowered the "perceived depth of relationship" with their GP.[21] In the hospital setting mortality and care may be related to high workload and job stress. In a survey of 6,536 physicians in the US engaged in direct patient care, quality of physician-patient communication was related to morale, job stress, time pressures and practice volume overload.[22] Work-hour regulations of interns introduced in 2003 were associated withdecreased short-term mortality among high-risk medical patientsin teaching hospitals but no association was found with mortality in surgical patients.[23] Similarly, the regulations were not associatedwith a change in mortalityfor Medicare patients in the first two years after implementation, but were in another analysis associatedwith significant relative improvement in mortality for patientswith four common medical conditions in more teaching-intensiveVA hospitals in postreform year two.[24]

Time spent on direct (face to face) patient care as a proportion of total workload did not predict practice performance. Similar results were reported by Murray, who found that part-time physicians (<40 hours per week) yielded similar patient evaluations of their performance compared to full-time working physicians (40–65 hours per week).[25] Using Europep as patient questionnaire Heje in Denmark found a negative association between list size and accessibility but not between list size and evaluation of GP-care.[26] It seems necessary to relate workload to list size to predict practice performance.

Earlier studies on workload and job stress were often done in countries with a largely capitation based system (UK, The Netherlands). The assumption was that rewarding equal practice size roughly rewards equal workload and job stress. Disadvantages of capitation remuneration were that workload differed per GP and experienced workload or job stress even more. Being a perfectionist for example, being salary paid or being less in control of the organisation (less autonomy) resulted in higher job stress.[27,28]

### Implications

#### For practice performance

A BMA report suggested that work-related stress amongst doctors must be addressed by reducing demands (by flexible employment practices and organizational climates discouraging excessive working hours), by increasing job control (by increasing staff participation in decision making) and, by increasing support to the individual (by ensuring good career and staff development strategies and promoting formal and informal social support).[29] Our study suggests that practices with lower than average practice performance should also look into actual GP time per patient and job stress. Only lowering the hours of direct care provided by GPs may not help to prevent burn out and stress and may prove counterproductive. Experts recommend feedback, coaching and supervision as means to deal with these problems.[30]

#### For policy

The strongly positive experiences with more time per patient (twice as much as in the US)reported by Australian and New Zealand adults indicate that having more time to spend with patients makes a difference.[31] In a previous study longer booking intervals for patient consultations proved to be of psychological advantage to general practitioners. Surgery sessions with patients booked at 10 minute intervals (experimental sessions) were compared with the doctors' usual booking intervals of between 7.5 and 5.0 minutes (control sessions).[32] In Spain "the 10 minutes platform" of concerned GPs demands a minimum of 10 minutes consultation time and they prefer 15 minutes intervals.

The Netherlands had a capitation fee system until 2007. This limits generalisability to fee for service systems.

#### Education

Well organised practices should collect data on practice performance and workload for organisational development and team communication using internationally validated instruments as in European Practice Assessment (EPA).[33] For team development, tools such as the Maturity Matrix may be helpful.[34]

#### Research

Practice performance is multifactorial and complex. Yet an international comparative study relating practice performance to consultation time, time per patient, indirect time spent on patient care and job stress using the practice visit method EPA would provide valuable information.[35]

A prospective study to analyse if practice performance improves by increasing practice time per patient would be valuable for future policy on manpower.

## Conclusion

More GP-time per patient and less job stress are related to better practice performance. The findings add weight to previous work on workload, which mostly focused on the physician rather than the practice as in our study. Workload and job stress should be monitored at the national, local and practice level using list size adjusted data. A prospective study could clarify a possible causative relation. Organisational development using feedback on workload and job stress could benefit both patients and GP.

## Competing interests

The authors declare that they have no competing interests.

## Authors' contributions

PvdH was the main author, developed the practice visit method, originated the research questions and wrote the paper. BK is writing a PhD on workload and job stress of GPs and contributed intellectually. He commented on each draft and the paper will be a chapter in his thesis. GE contributed using his knowledge of the international literature and he commented on each revision. JvD did all the data management and analysis. RA commented on the study design, supervised the data processing and analysis. RG was involved from the start of the development of the practice visit method, supervised the project and commented on the paper. MW was involved in developing the research questions and in supervising the analysis. He contributed much to the final draft. All authors read and approved the final manuscript.

## Pre-publication history

The pre-publication history for this paper can be accessed here:


